# Supplementary material for: Precision size and refractive index analysis of weakly scattering nanoparticles in polydispersions
Source: Nat Methods. 2022 May 9;19(5):586–93. doi: 10.1038/s41592-022-01460-z (PMC9119850; doi:10.1038/s41592-022-01460-z)
Supplement: Supplementary file 1 — Supplementary Figs. 1–18 and Discussion. [file 41592_2022_1460_MOESM1_ESM.pdf]

nature portfolio

https://doi.org/10.1038/s41592-022-01460-z

Supplementary information

Precision size and refractive index analysis of weakly scattering nanoparticles in polydispersions

In the format provided by the authors and unedited

Precision size and refractive index analysis of weakly scattering nanoparticles in polydispersions

Anna D. Kashkanova,[1,2†] Martin Blessing,[1,2,3†] André Gemeinhardt,[1,2,3] Didier Soulat[4]
and Vahid Sandoghdar[1,2,3*]
[1]Max Planck Institute for the Science of Light, 91058 Erlangen, Germany
[2]Max-Planck-Zentrum für Physik und Medizin, 91058 Erlangen, Germany
[3]Department of Physics, Friedrich-Alexander Universität Erlangen-Nürnberg, 91058 Erlangen, Germany
[4] Institute of Clinical Microbiology, Immunology and Hygiene, Universitätsklinikum Erlangen
and Friedrich-Alexander Universität Erlangen-Nürnberg (FAU), 91054 Erlangen, Germany
† Equal contributions
* Corresponding author. Email: vahid.sandoghdar@mpl.mpg.de

Contents

1 Sample preparation **2**
1.1 Measurement chambers . . . 2
1.2 Liposomes . . . 2
1.3 *Leishmania* EVs . . . 2
1.4 Human urine . . . 2

2 Measurement **3**
2.1 Relating iSCAT contrast to scattering cross-section . . . 3
2.2 Camera calibration . . . 5
2.3 Drift and vibration estimation . . . 6
2.4 Other measurement considerations . . . 6
2.5 Measurement time . . . 7
2.6 Reproducibility . . . 8

3 Analysis **8**
3.1 Video analysis . . . 8
3.2 Trajectory analysis . . . 9

4 Details of measurements on biological samples **11**
4.1 Analysis with a simple solid-sphere model . . . 12
4.2 Details of liposome measurements . . . 12
4.3 Details of human urine measurements . . . 12
4.4 Reproducibility of biological sample measurements . . . 16

1 Sample preparation

1.1 Measurement chambers

Coverslips (#1.5 VWR, Darmstadt/Germany) and gaskets (Grace Biolabs, Bend OR/USA) were cleaned in 2% Hellmanex III solution (Hellma, Muellheim/Germany), milliQ, Ethanol, and Isopropanol subsequently and finally blow dried with N_2 gas. A well was formed by pressing the gasket onto the coverslip. Next, particles of various sizes and materials were dissolved in milliQ. We set the concentration such that only a few particles are visible within the field of view. Finally, 60 µl of the particle suspension was added to the well, and it was closed with another coverslip to avoid evaporation.

1.2 Liposomes

The lipids DOPC (1,2-dioleoyl-*sn*-glycero-3-phosphocholine) and DOPS (1,2-dioleoyl-*sn*-glycero-3-phospho-L-serine [sodium salt]) were purchased from Avanti Polar Lipids Inc. (Alabaster, AL/USA), resuspended in chloroform and mixed together with molar fractions of 95% and 5%, respectively. The mixture was first blow-dried with nitrogen and desiccated subsequently for at least 30 min. The lipid film was resuspended in pure water to obtain a 1 mg/ml concentration. After incubation for at least 1 h at room temperature, the sample was thoroughly vortexed and transferred to a clean extruder, holding a 200 nm filter membrane. The sample was extruded over 40-60 cycles and transferred to an Eppendorf tube. For the iNTA measurement, the samples were diluted in milliQ to adjust the particle number in the field of view.

1.3 *Leishmania* EVs

Extracellular vesicle isolation was performed as described previously with minor modification.[1] Briefly, *Leishmania major* promastigotes (strain MHOM/IL/81/FEBNI)[2] were grown to stationary phase (*c.a.* 5.0×10^7 c/ml) in complete Schneider medium containing 10% fetal calf serum (FCS) at 28°C, 5% CO_2. Next, parasites were counted and centrifuged for 10 min at $1000g$ at room temperature to discard the growth medium. Parasites were then washed with PBS. Afterward, 7.5×10^8 parasites were transferred in 15 ml of RPMI cell culture medium complemented with penicillin (100 units/ml) and streptomycin (100 µg/ml) without FCS in order to avoid EV contamination. Promastigotes were incubated for 16 hours at 28°C, 5% CO_2. Next, parasite culture was centrifuged for 10 min at $1000g$ to remove parasites. The supernatant was then subjected to sequential centrifugation at $1000g$ (30 min) and $15000g$ (45 min) to remove dead parasites and debris. Additionally, the supernatant was passed through a 0.45 µm cutoff filter. Finally, the supernatant was centrifuged at $110000g$ for 1 hour (70 Ti rotor, fixed angle, Beckman Coulter) to pellet EVs. All centrifugation steps related to the EV purification were done at 4°C. EVs were finally resuspended in 100 µl sterile and filtered PBS. EVs were stored in PBS at 4°C until measurement. Right before the measurement, the sample was diluted in milliQ to adjust the particle number in the field of view.

1.4 Human urine

Without any additives for stabilization or preservation, fresh urine was collected from a healthy (no clinical or pathology diagnoses known), alive volunteer donor. The sample was centrifuged at $180g$ and 4°C for 15 min. The supernatant was immediately collected and centrifuged at $1550g$ and 4°C for 25 min. The resulting supernatant was shock frozen with liquid nitrogen and stored at -80°C. The urine samples were thawed on the day of each experiment.

In control measurements, urine samples were treated with unspecific protease and various types of detergents. As detergent, 2% Triton, 1% Octyl-beta-Glucoside, 0.6% Sodium cholate hydrate, and 0.4% CHAPS were mixed in DPBS (1x) and added to the urine sample in a 1:1 ratio. As an unspecific protease, we used proteinase K (Ambion Inc., Austin/US) at a final concentration of $200\,\mu\mathrm{g/ml}$) and incubated it at 37°C for 1.5 h. We also performed negative control experiments, where one urine sample was kept at 37°C for 1.5 h but no protease was added. Additionally, we measured a urine sample mixed 1:1 with DPBS. For the fluorescence measurement, urine treated with proteinase K was subsequently incubated with Octadecyl Rhodamine B Chloride (R18; final concentration: $2\,\mathrm{ng/ml}$) for at least 5 min without further purification.

According to Ref. 3, we performed western blots to identify EV-associated proteins, including two cytosolic proteins (Alix, TSG101), one peripheral (FLOT-1) and two integral membrane proteins (CD81, CD63). Furthermore, we identified a non EV-associated protein (UMOD). We emphasize that the used urine samples analysed in iNTA were not ultracentrifuged and proteins of interest were therefore hardly visible in the western blots, despite its high sensitivity. Consequently, we up-concentrated the sample by about 10-20x using Proteus X-Spinners 2.5 from Protein Ark (10kDa MWCO). We used Mini-Protean TGX precast gels (4-15%, 120V, 50min) together with the TransTurbo Blot and RTA Transfer Kit from Biorad Inc. As lysis buffer, we used Lämmli buffer plus β-mercapthoethanol (ratio 9:1). Primary and secondary antibodies are listed in Table 1.4. For TEM imaging, an equal volume of the urine sample was mixed with 4% paraformaldehyde solution.

Specificity	Manufacturer	Catalogue number	Clone No	LOT	dilution
anti-TSG101	Proteintech	28283-1-AP	NaN	00080154	1:2000
anti-Flotilin-1	Proteintech	15571-1-AP	NaN	00053543	1:3000
anti-Uromodulin	Proteintech	11911-1-AP	NaN	00056125	1:750
anti-CD63	Proteintech	67605-1-Ig	3D4D1	10015952	1:1000
anti-CD81	Proteintech	66866-1-Ig	1G2C6	10017531 and 10011817	1:1000 and 1:3000
anti-Alix 6	Proteintech	67715-1-Ig	1H9D9	10017670	1:2000
anti-Mouse IgG	Bio-Rad	170-6516	NaN	64322731	1:10000
anti-Rabbit IgG	Bio-Rad	170-6515	NaN	64332296	1:10000

To demonstrate that the vesicles are still intact after proteinase K treatment, we added the protease also in our western blot experiments with increasing concentrations (0.5,1,2,4 and $6\,\mu\mathrm{g/ml}$). The samples were incubated at 37°C for 90 mins. Subsequently, we added PMSF with a final concentration of 7.5 mM and incubated it for another 30 min at ambient temperatures before adding the lysis buffer.

2 Measurement

2.1 Relating iSCAT contrast to scattering cross-section

The intensity detected in a reflection iSCAT setup results from the interference between the reflected electric field with intensity $|E_{\mathrm{ref}}|^2 = r^2|E_{\mathrm{inc}}|^2$ and the scattered field with intensity $|E_{\mathrm{scat}}|^2 = |s|^2|E_{\mathrm{inc}}|^2$. Here, r is the amplitude reflectance coefficient of the substrate, and the scattering efficiency s^2 scales with the particle scattering cross-section.

$$I_{\mathrm{det}} = |E_{\mathrm{ref}} + E_{\mathrm{scat}}|^2 = |E_{\mathrm{inc}}|^2 \left(r^2 + |s|^2 + 2r|s|\cos\phi\right) \tag{1}$$

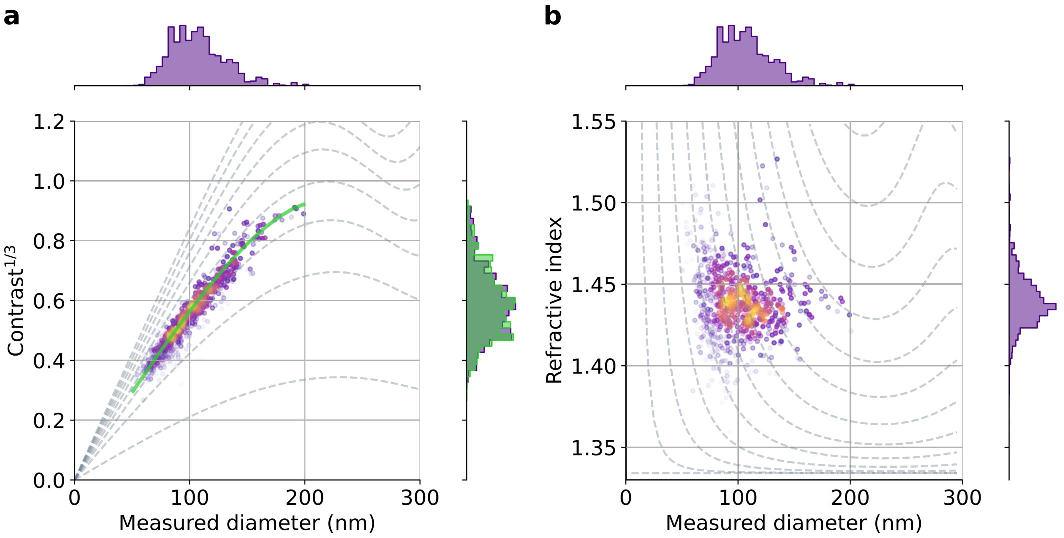

Figure S1: Calibration using 100 nm silica beads (a) Scatter plot for the third root of particle contrast ($\sqrt[3]{C}$) and the measured particle size. The dashed lines denote solid spheres with different refractive indices, increasing from 1.34 to 1.66 in steps of 0.04. **(b)** The extracted refractive index of the particle plotted vs. its size. The dashed lines show $\sqrt[3]{C}$ from 0 to 1.2 in steps of 0.1.

The angle ϕ signifies the phase difference between the scattered and the reflected fields, and it depends on the relative position of the interface, the focal plane, and the scatterer.

The particle iSCAT contrast is defined as the ratio of the signal to the background $|E_{\text{ref}}|^2$, which amounts to

$$C = \left(\frac{|s|}{r}\right)^2 + 2\frac{|s|}{r}. \tag{2}$$

This expression can be rephrased as

$$C = \beta^2\sigma_{\text{scat}} + 2\beta\sqrt{\sigma_{\text{scat}}}\,, \tag{3}$$

where σ_{scat} is the particle scattering cross-section, and β is a proportionality constant that absorbs the ratio between s and $\sqrt{\sigma_{\text{scat}}}$ as well as $1/r$. Importantly, β only depends on the setup parameters. Therefore, as long as the setup does not change, we only need to determine it once in a calibration procedure.

We remark that we use the full Mie theory[4] to calculate the differential scattering cross-section of particles with diameter $\geq \lambda$. We then integrate the result to obtain the back-scattered light. Since we use a microscope objective with $NA = 1.46$, we assume that we collect all of the backward scattered light.

In the main manuscript, we use the measurements of particles of known size and refractive index to determine $\beta \simeq 3 \times 10^7\ \text{m}^{-1}$ for the focal plane located at 1 µm above the cover glass. To verify the calibration of the iSCAT contrast, we consider a careful measurement of 100 nm silica spheres. Figure S1 shows a 2D scatter plot for $\sqrt[3]{C}$ and d. We find a broad size distribution determined from the analysis of the particle trajectories. The histogram of the contrast also looks broad, but the occurrences on the 2D plot follow a clear characteristic trend. To understand this behavior, we plot the contours corresponding to various indices of refraction for silica. We find that the measurements are remarkably tightly confined between the contours corresponding to $n = 1.42$ and $n = 1.46$, consistent with the range of previously reported values for silica.[5,6]

Furthermore, we plot the green contour ($n = 1.44$), which goes through the center of the data cloud. If we now assume this refractive index for silica and the measured size distribution given by the histogram on the upper axis, we

obtain the green histogram overlaid on the histogram of the measured $\sqrt[3]{C}$ displayed on the right-hand vertical axis. The remarkable agreement between the two (green and purple) histograms shows that the distribution in C is not dominated by measurement error, and that it is rather governed by the size distribution in the sample. Figure S1b uses the same data to display the measured distributions as a function of the refractive index and diameter. The excellent agreement of the measured data with Mie theory provides a robust calibration that we use for unknown samples such as extracellular vesicles discussed in the last section of the manuscript.

We note that considering the possibility of varying phase angle ϕ, particles of different size can result in the same iSCAT signal if one monitors the destructive interference term. To avoid this ambiguity, we use the the maximum positive interference contrast, i.e., when $\cos\phi = 1$.

2.2 Camera calibration

In order to extract the iSCAT contrast correctly, the camera response function needs to be considered. It can be estimated by sending a known amount of light onto the camera and measuring the average pixel count of a uniformly illuminated FOV. The outcome of this calibration, along with an interpolating function is shown in Fig. S2(a). It is clear that the result is non-linear, especially for counts below 1000. Therefore, to calculate C correctly, we need to convert each pixel value from the camera counts to the power landing on the camera. Note that if one does not perform this calibration and instead, subtracts the "dark frame" (frame recorded without incident light), the iSCAT contrast might become underestimated by up to 3 times.

Figure S2(b) deals with another important question, namely, the maximum $\sqrt[3]{C}$ that can be measured in a setup before the camera is saturated. This depends on the mean camera counts in a given frame. The power values used in most of the experiments corresponded to 2 mW, 4 mW, 10 mW, and 40 mW measured before the objective, leading to the maximum $\sqrt[3]{C}$ values $\simeq 3$, 2.3, 1.6, and 0.9, respectively.

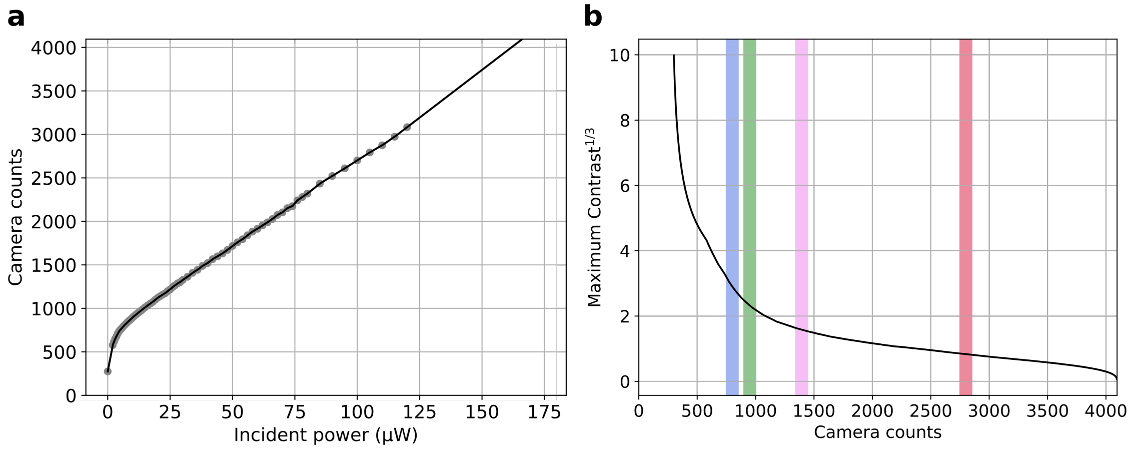

Figure S2: The power calibration of the camera. **(a)** The mean camera counts recorded vs. the power incident on the camera together with an interpolating function. **(b)** Maximum measurable $\sqrt[3]{C}$ as a function of the camera counts. The shaded areas indicate the approximate ranges of camera counts in the experiments performed at 2 mW (blue band), 4 mW (green band), 10 mW (violet band), and at 40 mW (red band) laser power.

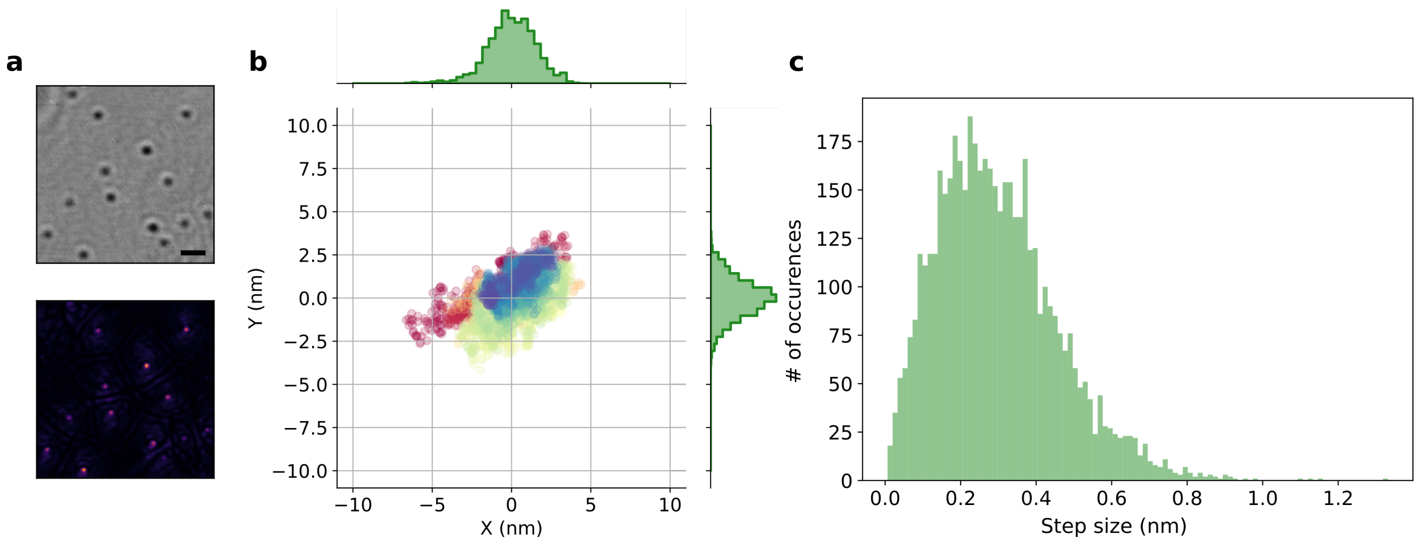

Figure S3: (a) Image of the structured cover slide after background correction (top) and RVT operation (bottom). Scale bar is 1 µm. (b) Average of trajectories of 8 substrate indentations, together with the histograms. (c) Absolute step size distribution.

2.3 Drift and vibration estimation

To estimate the drift and vibration of the setup we used a sample with etched indentations, similar to the one used in Ref. 7. The sample is mounted on the setup and allowed to settle for a few minutes. Several 1 s long videos are then recorded at 5 kHz. The examples of a background corrected frame and RVT applied to the frame are shown in Fig. S3(a). We track 8 indentations individually and then average the trajectories to extract the motion of the cover glass. The average trajectory recorded over 1 s is shown in Fig. S3(b), whereby color indicates temporal order. The maximum range of motion is about 10 nm. Finally, in Fig. S3(c) we show the absolute step size that the cover glass takes between two consecutive frames. The median value is below 0.3 nm.

2.4 Other measurement considerations

For particles with a small scattering cross-section, a larger power is needed to achieve a sufficient SNR. On the other hand, the illumination power should be kept limited for particles with large scattering cross-sections because radiation pressure can push the particles axially.[8,9] Therefore, it could be beneficial to compare measurements performed at two different powers. Another issue to consider concerns gravitational forces, which can affect denser particles, leading to sedimentation. This effect makes the background noisier and the measurements more challenging.

Figure S4a shows the comparison of measurements performed at 40 mW and 4 mW for liposomes. Here the low power measurement almost completely misses the population. Figure S4b displays the outcome of *Leishmania* EVs measurement performed at 40 mW and 2 mW, and Fig. S4c displays the outcome of urine EVs measurement performed at 40 mW and 4 mW. In all three cases, it is evident that measurements at higher illumination power give access to lower contrasts and, thus, to smaller particles and/or particles with lower indices of refraction.

In order to achieve a homogeneous illumination for camera calibration, an acousto-optical deflector (AOD, Opto Electronic, France/Orsay) was positioned right after the laser and used to scan the beam transversely at a frequency of 100 kHz. However, an AOD is not necessary for the measurements presented in this work. All experiments could also be performed in a conventional wide-field iSCAT setup.

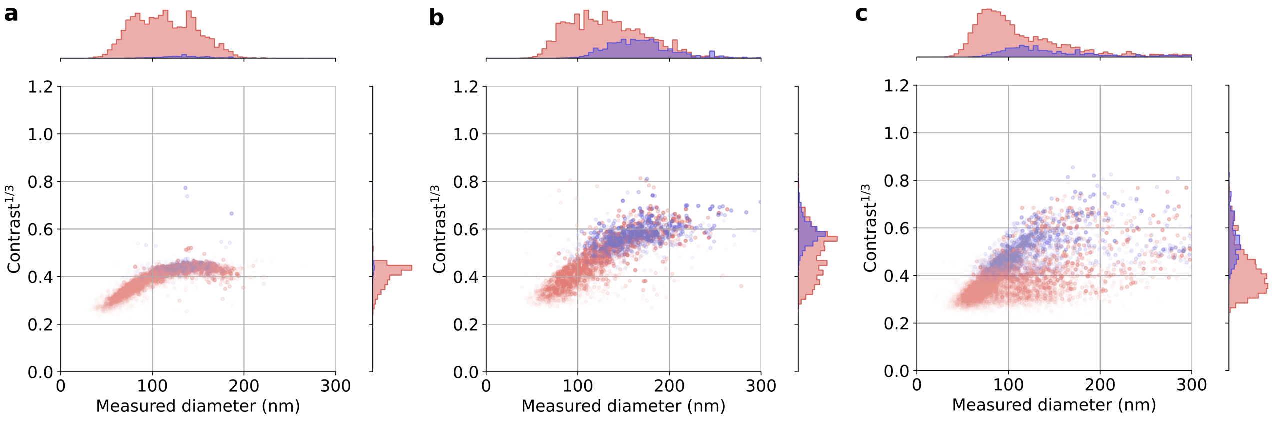

Figure S4: Measurements of mixtures at different powers. Scatter plots of the third root of iSCAT contrast $\sqrt[3]{C}$ and the measured particle size. The dashed lines correspond to different effective refractive indexes, ranging from 1.34 to 1.66 in steps of 0.04. The data taken at 40 mW are shown in red, while the data taken at 4 (2) mW are shown in blue. The samples are **(a)** liposomes recorded at 40 mW and 4 mW **(b)** *Leishmania* EVs recorded at 40 mW and 2 mW and **(c)** human urine recorded at 40 mW and 4 mW.

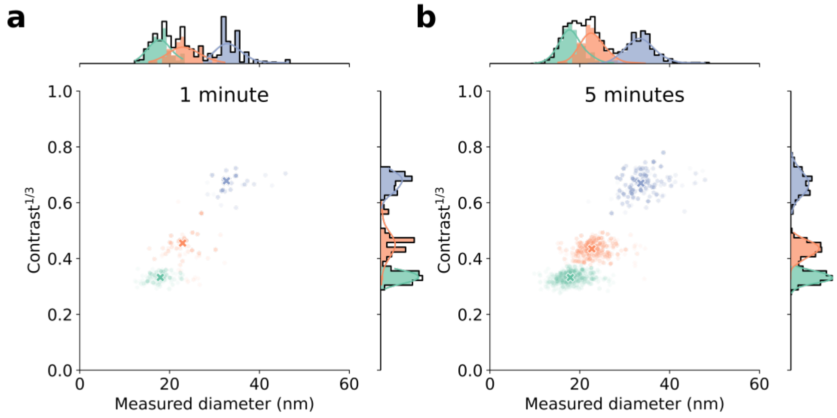

Figure S5: Measurement of a mixture of 15 nm, 20 nm, and 30 nm GNPs for different measurement times.

2.5 Measurement time

The measurement time is determined by the size of the signal and by the desired precision and accuracy: the weaker the signal or the higher the desired resolution, the longer is the required data collection time. For more concentrated monodisperse samples, a few seconds of measurement is sufficient for iNTA. For example, the data presented in Fig. 2d of the main manuscript was recorded over 5 seconds. For more complex polydisperse sample and for samples with low particle concentration, longer measurements are helpful. The data presented in Fig. 3 and Fig. 4 of the main manuscript were recorded over 10-20 minutes. In Fig. S5, we show the outcome of the first 1 or 5 minutes of the measurement presented in the Fig. 3c of the manuscript to demonstrate the evolution of the data quality as a function of the measurement time. A comparison of Fig. S5b and Fig. 3c of the manuscript clearly shows that the improvement obtained between 5 min and 20 min is incremental for deciphering the three populations. However, the longer the measurements, the more reliably can one recognize weak features, which might be, e.g., indicative of a disease or rare species. The measurements performed with commercial NTA used in this paper were all performed over 5 minutes.

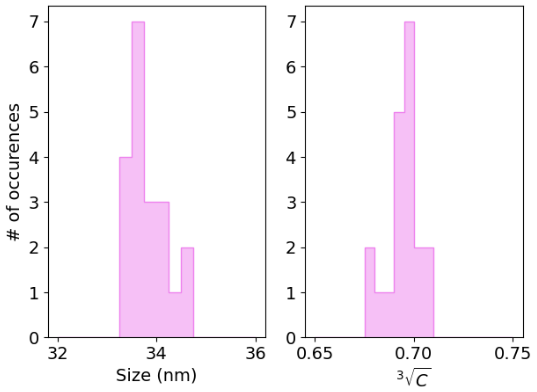

Figure S6: Size and third root of iSCAT contrast for twenty 30 nm samples measured over the course of two weeks.

2.6 Reproducibility

To address the question of reproducibility, we measured twenty identical 30 nm GNP samples over the course of two weeks. The focal plane was always positioned 1 μm above the coverglass. The results are shown in Fig. S6. The mean value of the particle size amounts to 33.8 nm with standard deviation of 0.4 nm. The mean value of the third root of the iSCAT contrast is 0.69 with standard deviation of 0.01.

3 Analysis

3.1 Video analysis

The analysis procedure is as follows. First, we use the calibration described in section 2.2 to correct the raw frames by converting the pixel counts to power incident on the camera. This is necessary because the camera response is non-linear. Afterwards, we calculate the temporal median frame for the whole movie (5000 frames) and subtract it from each corrected raw frame. That renders the particles significantly more visible, however makes the camera read-out ("column") noise very prominent. We remove the column noise in each frame by subtracting from each column its median. We add the median of the whole image to keep the average pixel value of the image constant. Finally, we apply normalized Radial Variance Transform (RVT)[7] to transform each iPSF into a bright, easily trackable spot. The variance ratio filter is then applied. For each pixel it calculates the variance of the pixels in its neighborhood $r < r_{\max}$ and the variance of the pixels in the "doughnut" neighborhood $r_{\min} \leq r < r_{\max}$. After that, each pixel in the image is multiplied by the ratio of these two variances. This filter highlights sharp peaks produced by RVT and removes the smooth background. We track particles on a video of filtered RVT images using Python TrackPy package.[10] We only keep the trajectories with more than 25 localizations.

We convert each background-corrected image to a "contrast" image by dividing it by the temporal median frame. Then, we generate a Gaussian mask with the width equal to 1 pixel at the subpixel location of the original particle. We multiply the contrast image by the mask. The contrast of the particle is defined as the sum of the pixels in the multiplied image, divided by the sum of the pixels in the mask multiplied by a Gaussian of width $s_0 \simeq 80$ nm, which is the width of the Gaussian approximation of the PSF (for our parameters of wavelength $\lambda = 520$ nm, NA = 1.46). Choosing a different value of s_0 simply scales the value of the calibration constant β.

In each trajectory, we find the localization at the maximum positive contrast. For this localization, we additionally

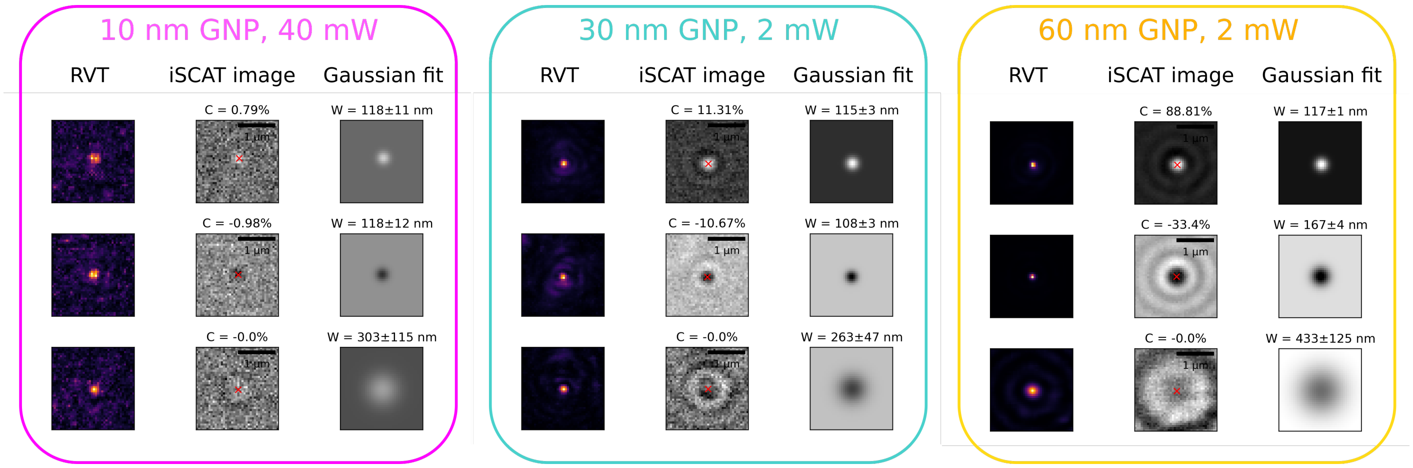

Figure S7: Some of the analysis steps for selected iPSFs. In each box, the middle column shows the iSCAT image after temporal median background subtraction. The left column shows the RVT images which are fit to extract the iPSF center. The right column shows the results of a 2D Gaussian fit to the center of the iPSF shown in middle column. The width of the fitted Gaussian (given here in nm) is monitored to identify the position (moment), when the particle crosses the focal plane. Each box also shows 3 rows for 3 iPSFs extracted from a single particle trajectory. The top row displays the iPSF with maximum positive contrast, the middle row shows the iPSF with maximum negative contrast, and the bottom row plots the iPSF with the minimal absolute contrast. The contrast values are indicated above the iPSFs in the middle column.

perform a fit of a Gaussian to the central feature of the iPSF to check whether the particle is located near the focal plane. Near the focal plane the width of the fitted Gaussian is minimal. For the focal plane located 1 µm above the cover glass, we find that the iPSFs, whose central lobe can be fitted with a Gaussian of a width between 100 nm and 130 nm correspond to the particles crossing the focal plane.

Figure S7 presents a few examples of the iPSFs for GNPs of various sizes. It also shows the corresponding RVT images used to localize the iPSF centers as well as Gaussian fits to the iPSF centers.

3.2 Trajectory analysis

Each video is recorded over 1 s at 5 kHz. Figure S8 shows examples of the extracted trajectories for different particles and time intervals. For each trajectory, we calculate the mean squared displacements (MSD) for different time steps between 1 frame (0.2 ms) and 25 frames (5 ms). Following Ref.,[11] the MSD vs. time plot can be fit to a function given by

$$\mathrm{MSD}(t) = a + bt. \tag{4}$$

Assuming that 2D projections of 3D trajectories are used, one can extract the diffusion constant as

$$D = b/4 \tag{5}$$

and the static localization error as

$$\sigma_{\mathrm{Loc}} = \frac{1}{2}\left(a + \frac{bt_{\mathrm{exp}}}{3}\right)^{1/2}, \tag{6}$$

where t_{exp} is the camera exposure time.

When choosing the number of points in the MSD plot to include in the fit, one should consider two effects: 1) localization errors add noise to the MSD plot for short time lags so that fitting too few points will result in erroneous

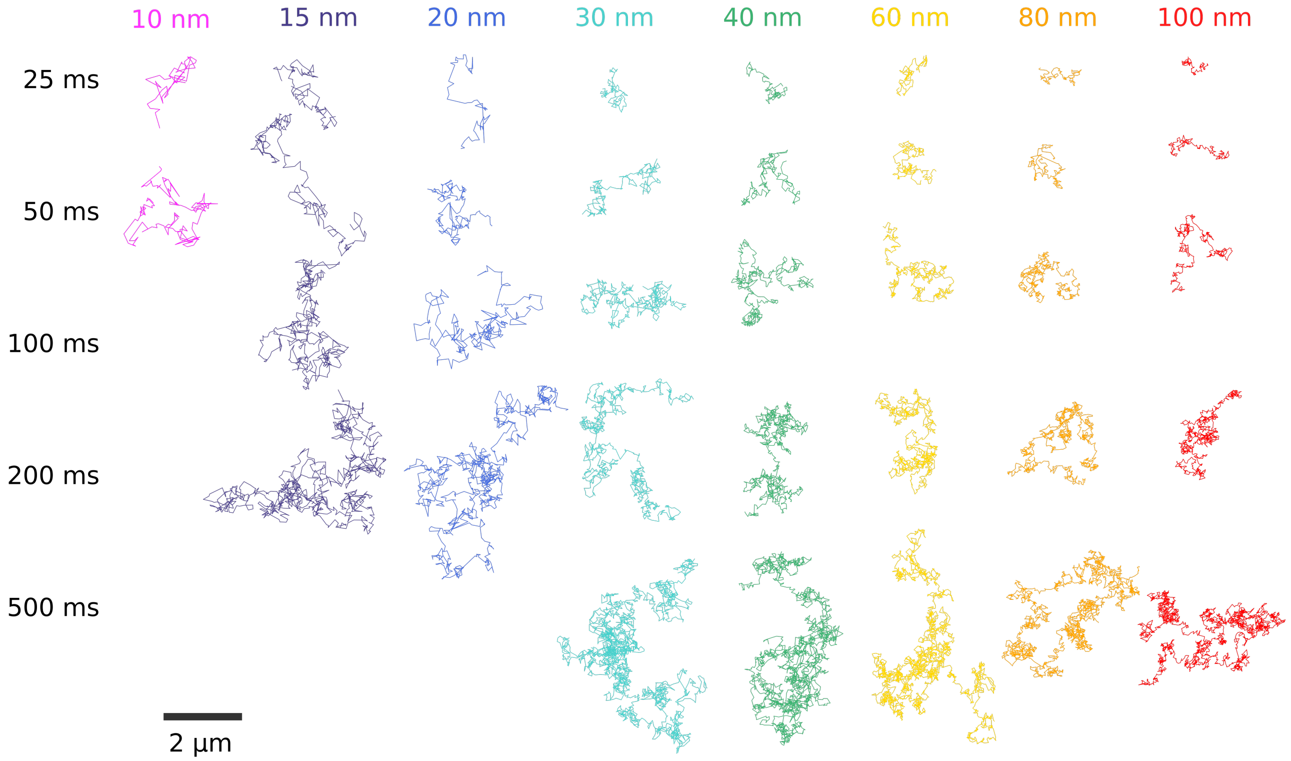

Figure S8: Sample trajectories of different sized GNPs over different intervals of time. For smaller particles there are no trajectories above a certain length as they diffuse out of the observable volume too fast.

diffusion constants, and 2) at large time lags, the relative standard deviation of the MSD becomes larger since fewer squared displacements are averaged. Therefore, there is an optimal number of points to fit. When the localization error is known, one can estimate the optimal number of points using an empirical expression given in Ref. 11,

$$p_{\mathrm{opt}} = 2 + 2.7\sqrt{x} \tag{7}$$

with the trivial condition that $p_{\mathrm{opt}} < N$, where N is the number of localizations in a trajectory. Here $x = \sigma_{\mathrm{loc}}^2/D\Delta t$ is the reduced localization error, where Δt is the time between frames.

In our case, the value of x is unknown, but we have many trajectories recorded for particles of nominally the same size and diffusion constant. Therefore, we approach the problem differently. We start by fitting MSD of each individual trajectory over (2, 3,..., 25) points and extract the corresponding diffusion constants (D_2, D_3,..., D_{25}). This leaves us with 24 arrays of diffusion constants, one for each possible number of points. Then we determine the number of fit points, for which the weighted (by trajectory length[12]) relative standard deviation of the array is minimized and declare that to be optimal. We note, however, that choosing any other number of points does not affect the extracted D by more that 5%.

The localization errors are calculated using Eq. 6 to be about 70 nm for the smallest particles (10 nm GNP), which move by about $\sqrt{4Dt_{\mathrm{frm}}} \simeq 150\,\mathrm{nm}$ in $t_{\mathrm{frm}} = 0.2\,\mathrm{ms}$ between two neighboring frames. Correspondingly, we find 9 nm for the largest particles (100 nm GNP), which move by about $\sqrt{4Dt_{\mathrm{frm}}} \simeq 60\,\mathrm{nm}$ in t_{frm}.

Since the particle can move during a single exposure, the iPSF becomes blurred. This is referred to as "motion blur". According to Ref. 11, motion blur should increase the particle localization error by a factor of $\sqrt{1 + \frac{Dt_{\mathrm{exp}}}{s_0^2}}$. For 10 nm GNPs, motion blur enlarges the localization error by less than 20% when particles are in focus. The effect is reduced when

particles are out of focus (since s_0 is larger), and for larger particles (since they diffuse slower). We note that uncorrelated localization errors and motion blur have no effect on the mean value of D and only affect the width of its distribution.[11]

4 Details of measurements on biological samples

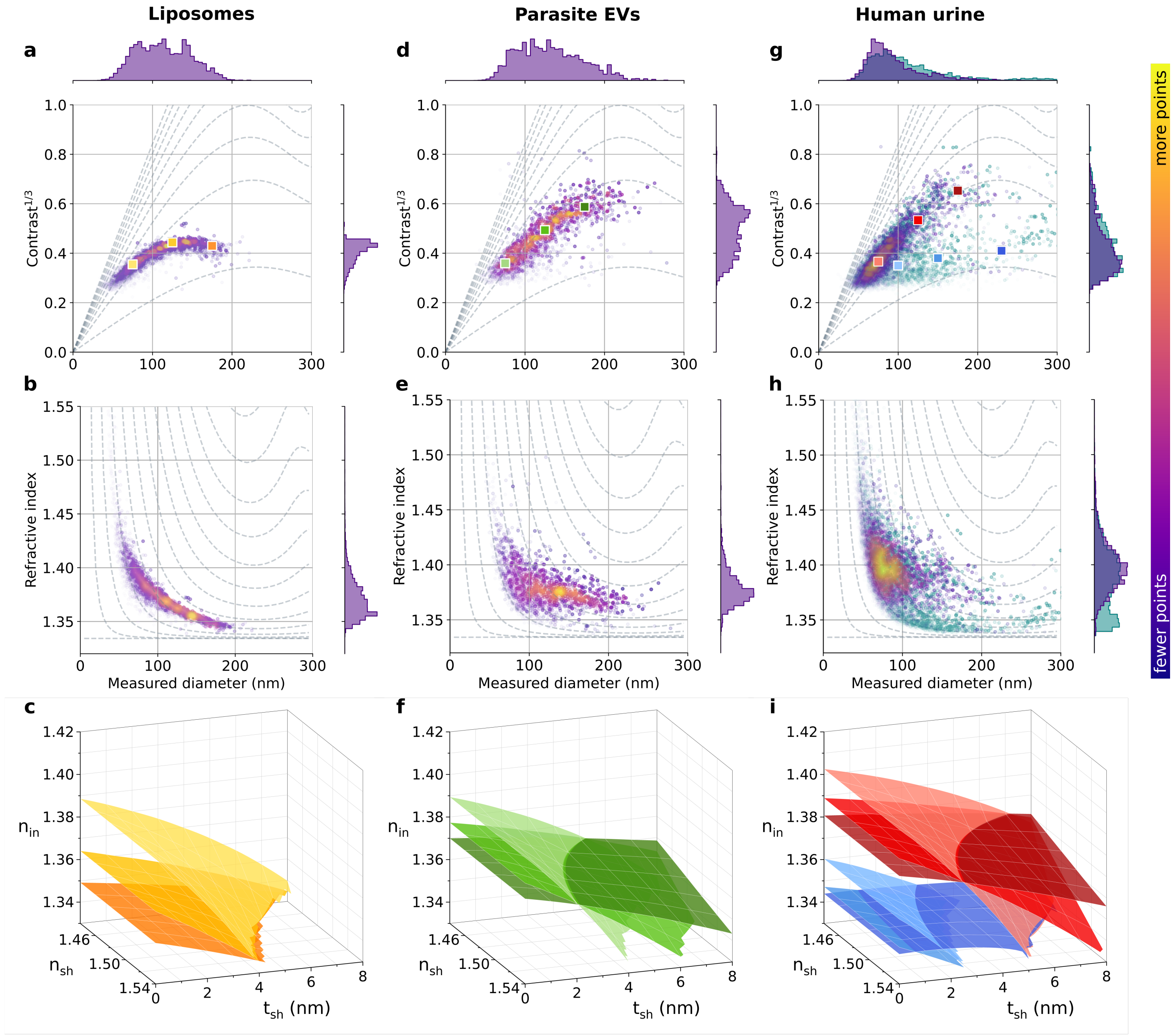

Figure S9: Application of solid sphere model to lipid vesicle samples The top row shows scatter plots for $\sqrt[3]{C}$ and extracted particle size. The dashed lines correspond to different refractive indices of the solid sphere, increasing from 1.34 to 1.66 in steps of 0.04. In the middle row, we show the scatter plot of the same data for the effective refractive index of the particle and its extracted size. The dashed lines show $\sqrt[3]{C}$ from 0 to 1.2 in steps of 0.1. The bottom row shows isosurfaces of constant iSCAT contrast and size for the points marked in the top row over a range of values for n_{in}, n_{sh} and t_{sh} . The samples are synthetic liposomes **(a, b, c)** *Leishmania* EVs **(d, e, f)**, and human urine **(g, h, i)**. All measurements were performed at 40 mW.

4.1 Analysis with a simple solid-sphere model

To contrast the success of our model of a "shelled particle" in describing our vesicle data, we re-plotted the results of Fig. 4 in the main manuscript with RI contour lines based on a solid-sphere Mie model. In Fig. S9a, it is evident that the contour lines cross the data cloud instead of following it (as was the case for the shell model in Fig. 4a of the main manuscript). To elucidate the deduced values of RI further, we show in Fig. S9b the same scatter plot for RI and size distributions. The effective RI varies with size from 1.4 for small liposomes down to 1.34 for large liposomes due to a change in the volume ratio between an outer membrane and an aqueous interior.

Figure S9 d,e,g,h also shows the corresponding plots for *Leishmania* and urinary EVs, respectively. Again, we see that the RI contour lines for a solid sphere model do not follow the trend of the data clouds. Interestingly, the detergent- and protease-sensitive subpopulation also cannot be described by a solid-sphere Mie model, which further strengthens our suspicion that these consist of sparsely formed protein aggregates (for more details see also 4.3).

In order to compare and benchmark the outcome from the solid-sphere model with that of the shell model, we investigated the theoretical calculations for the shelled-model particle by allowing the shell thickness to approach zero. The bottom row in Fig. S9 shows the outcome, which is the extension of Fig. 4 b,d,f in the main manuscript to $t_{\mathrm{sh}} = 0$.

4.2 Details of liposome measurements

To showcase the superior performance of iNTA, we compare our data with those of DLS and NTA. A sample of extruded liposomes was measured with DLS (ZetaSizer ZS) and NTA (Nanosight NS500), and iNTA. The first graph in Fig. S10a shows the results of DLS measurements (number-weighted distributions). The middle graph in Fig. S10b presents a very broad intensity distribution obtained in an NTA experiment. This is to be contrasted with the high-definition iNTA plot on the right side in Fig. S10c. On the last graph the NTA (gray) and DLS - Record 3 (yellow) size distributions are overlaid with iNTA size distribution. The green line indicates a fit to Mie theory.

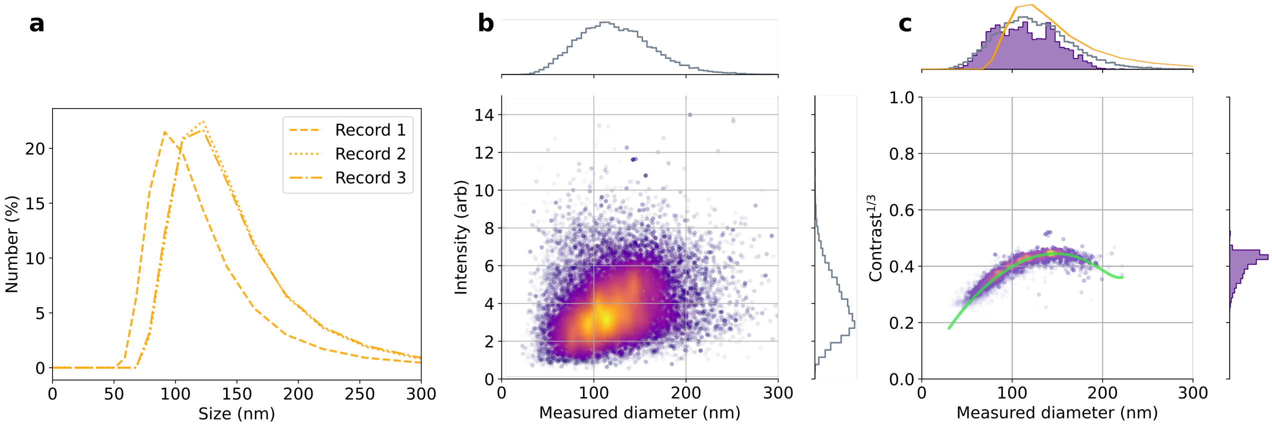

Figure S10: Characterization of liposomes extruded through 200 nm filter (a) with DLS (number-weighted), **(b)** NTA, or **(c)** iNTA.

4.3 Details of human urine measurements

Figure S11 shows additional iNTA measurements performed on urine samples treated with proteinase K (panel b) and a detergent buffer (panel d). Control measurements without protease (panel a) or detergent (panel c) are also shown. Both treatments (protease and detergent) changed the iNTA profile significantly, while the control measurements show no

changes. Based on these measurements, we identified two subpopulations: a protease- and detergent-sensitive population and a detergent-sensitive and protease-resistant population.

To further elucidate the nature of these subpopulations, TEM measurements were performed with untreated urine samples (see. Fig. S12). We identified both vesicular and filamentous features; the former dominated the image. Next, we performed western blots (WB) and detected five EV-associated proteins, comprising membranous and cytosolic markers. The whole WB membranes are shown in Fig. S13 and S14. Furthermore, we show that under proteinase K treatment cytosolic proteins are not digested. Only bands of the outer membranous markers (CD81, CD63) start to vanish with increasing proteinase K concentrations. We, therefore, conclude that the detergent-sensitive and protease-resistant population represents intact urinary extracellular vesicles. In Fig. S15, we also detected the non EV-associated protein uromodulin (UMOD) which is known to form filamentous protein complexes similar to the one identified in the TEM images. It stands to reason that the population that is sensitive to protease- and detergent, traces back to self-aggregates of uromodulin. The low effective refractive index of ≤ 1.35 (see also Fig. S9) would also be in agreement to its occurrence as fibril matrix.[13]

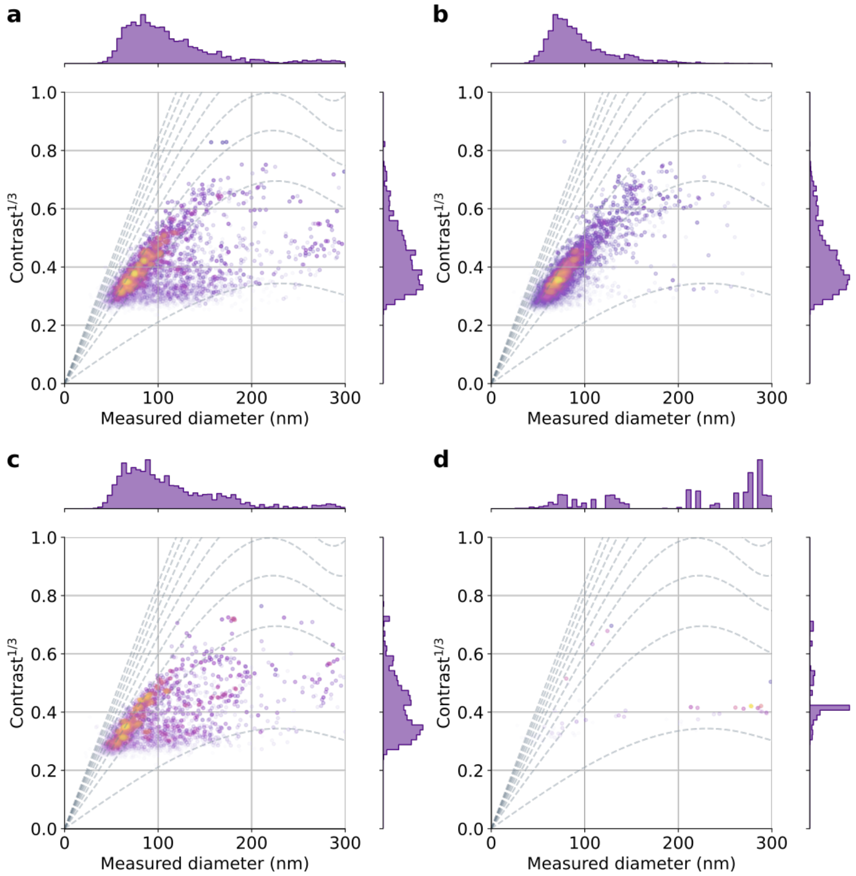

Figure S11: Protease and detergent treatment of urine. Urine incubated without **(a)** and with **(b)** proteinase K at 37°C for 1.5 hours. Urine mixed 1:1 with **(c)** DPBS or **(d)** detergent buffer

Additonally, we performed measurements in which urine treated with proteinase K was fluorescently labeled with a lipophilic dye (R18). The iSCAT and fluorescent measurements were performed simultaneously on two aligned and synchronized cameras. The cameras were aligned using spin-coated particles and synchronised using a signal output by the iSCAT camera as a trigger to the fluorescent camera. In order to prevent bleaching of the fluorescence, a shutter was employed to block the laser for 7 seconds and expose it for 3 seconds. In addition, the laser power was reduced. To collect

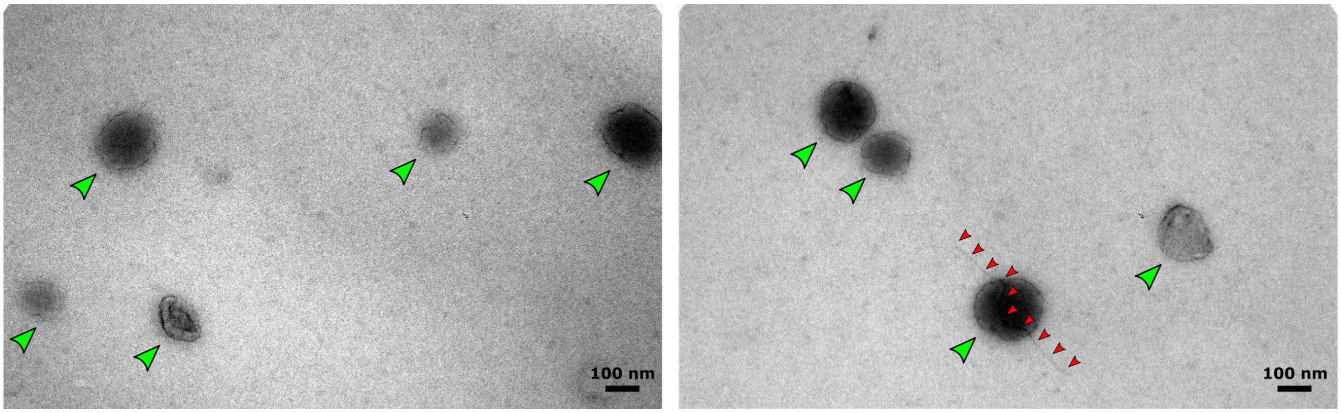

Figure S12: Characterization of urine with TEM. EVs of different characteristics can be identified (green arrows). A case of THP (UMOD) complexes is labeled with red arrows. The images are selected from a set of 61 images recorded during a single TEM measurement.

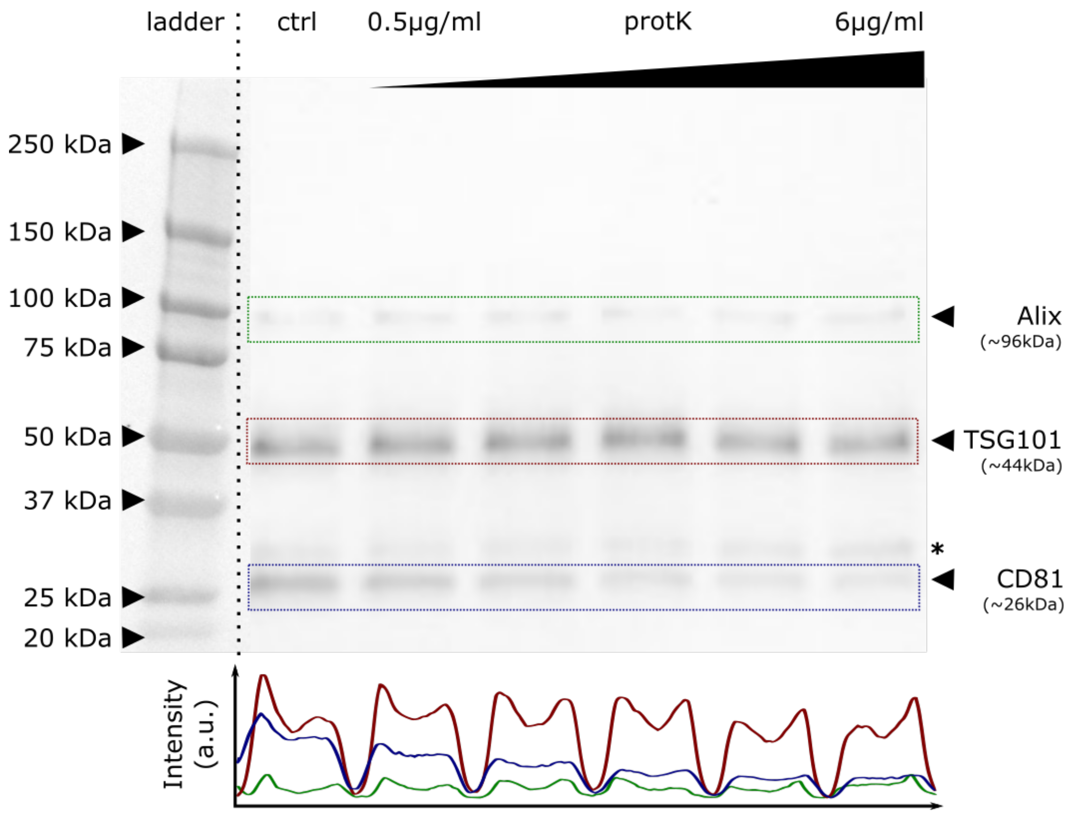

Figure S13: Western blot of up-concentrated urine sample detecting the cytosolic proteins Alix and TSG101, plus the integral protein CD81 (dilution 1:1000). Faint bands (asterisk) most likely represent palmitoylated CD81.[14] Samples were treated with (protK) or without (ctrl) proteinase K. The band intensity profiles of the respective proteins are displayed.

enough fluorescent photons, the frame rate was reduced to 1 kHz with 0.92 ms exposure time. The fluorescence video was median background corrected. Afterwards, at each position of the particle a value for fluorescence was extracted by using a small Gaussian mask, similar to the one used in the iSCAT analysis. The particle fluorescence was then assigned as the median of the trajectory's five highest fluorescence values. Fluorescence of each particle was then normalized by the standard deviation of the fluorescence video. Three exemplary iSCAT and fluorescence frames are shown in Fig. S16a. Some particles are marked along with their fluorescence values. We note that many particles were not showing

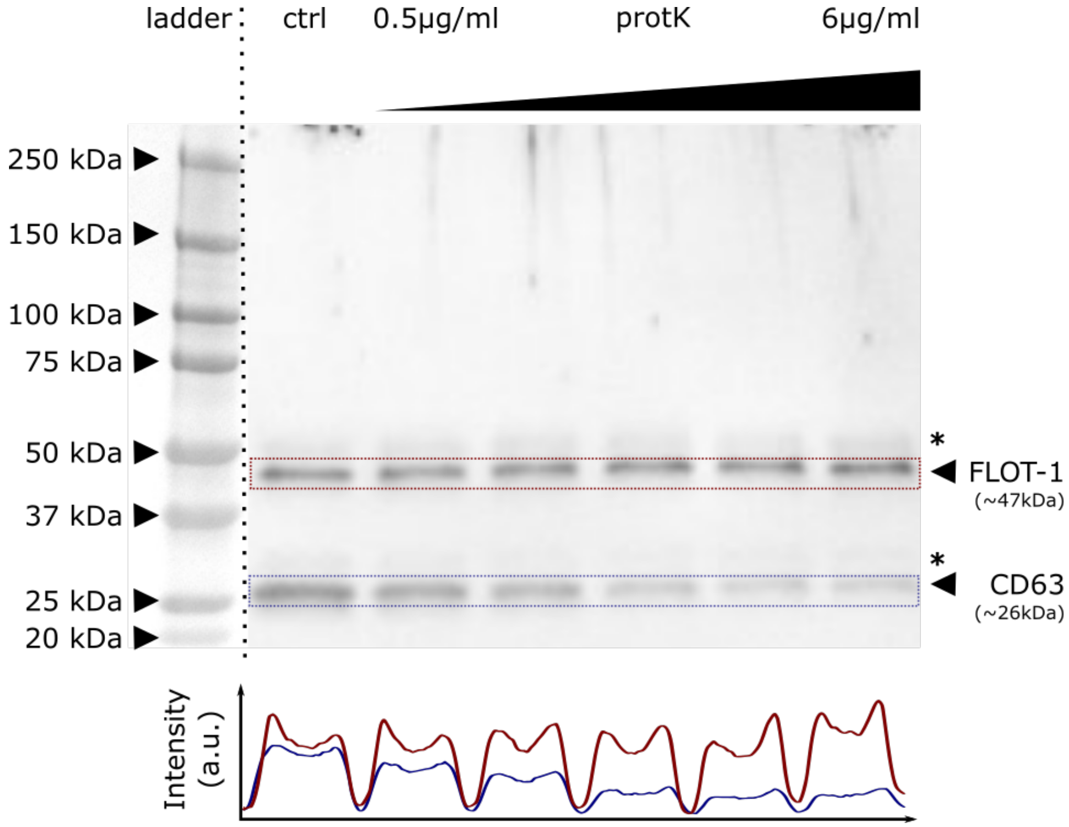

Figure S14: Western blot of up-concentrated urine sample detecting the peripheral membrane protein Flotilin-1 (FLOT-1), plus the integral protein CD63. Faint bands (asterisk) most likely represent palmitoylated Flotilin-1[15] and polylactosamined CD63,[16] respectively. Samples were treated with (protK) or without (ctrl) proteinase K. The band intensity profiles of the respective proteins are displayed.

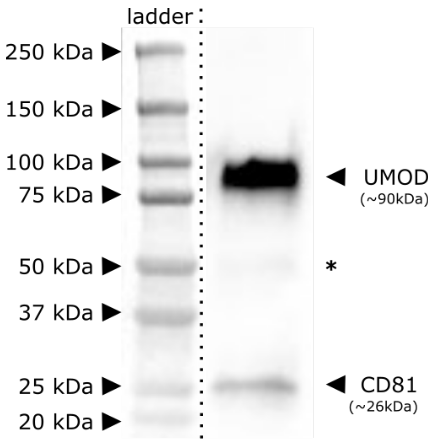

Figure S15: Western blot of up-concentrated urine sample detecting the non EV-associated uromodulin (UMOD) plus the integral protein CD81 (dilution 1:3000). Faint band (asterisk) most likely represent degraded uromodulin.

fluorescence. That could be caused by a low labeling efficiency or bleaching. In Fig. S16b, we show all particles tracked in an iSCAT video, while in Fig. S16c we only show particles with a fluorescent signal larger than 2σ above the video mean. It is evident that while the number of fluorescent particles is lower, the overall shape of the distribution remains unchanged.

Finally, we compared the performance of iNTA and NTA (Nanosight NS500) on two samples: urine incubated at 37°C

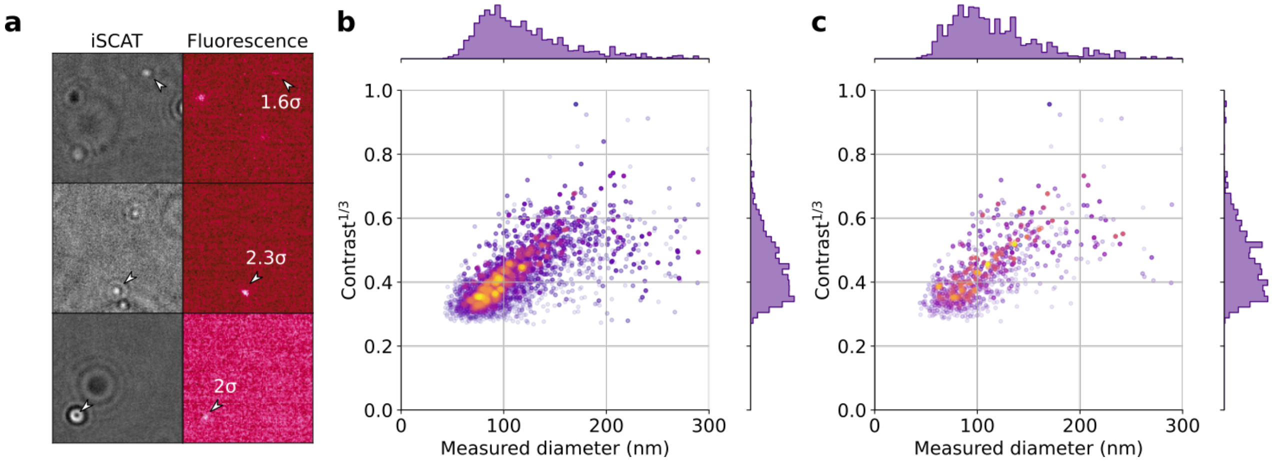

Figure S16: Measurement of fluorescently labeled urine. **(a)** Exemplary iSCAT and fluorescence frames. Arrows point out some of the particles together with their fluorescence values. Overall, 1 000 000 frames were recorded in both iSCAT and fluorescent channels. Here we show three of those.

(b) An iNTA plot of all particles present in urine **(c)** An iNTA plot consisting of particles with a fluorescence signal at least 2σ above the mean.

and urine treated with proteinase K. We performed measurements on Nanosight at three different camera gains (same as used in (5). The results are presented in Fig. S17. While iNTA reveals the difference between the two samples, NTA can not resolve it regardless of the camera settings used: the NTA data clouds in the top and bottom rows are nearly identical. Interestingly, NTA also performs worse on the urine sample compared to liposome sample (see Fig. S10). This is most likely caused by a larger variation in scattering cross-section of particles in urine, leading to bright particles saturating the field of view as the gain increases before the dim particles can be tracked. Indeed, the much smaller dynamic range needed for iSCAT (signal $\propto d^3$) as compared to dark-field microscopy (signal $\propto d^6$) is an intrinsic advantage of iNTA.

4.4 Reproducibility of biological sample measurements

In order to evaluate the reproducibility of the measurements performed on vesicles, we independently prepared multiple liposome and *Leishmania* EV samples as described in section 1. For urine, we collected several samples during a day and treated them in accordance to the protocol of section 1.4. Over the course of days, the samples were measured by iNTA either after freshly diluting the cooled stocks (liposomes, *Leishmania* EVs; both at 4°C) or thawing aliquots (urine samples). We then fit the data with the shell model. Figure S18 shows several examples of the measurements.

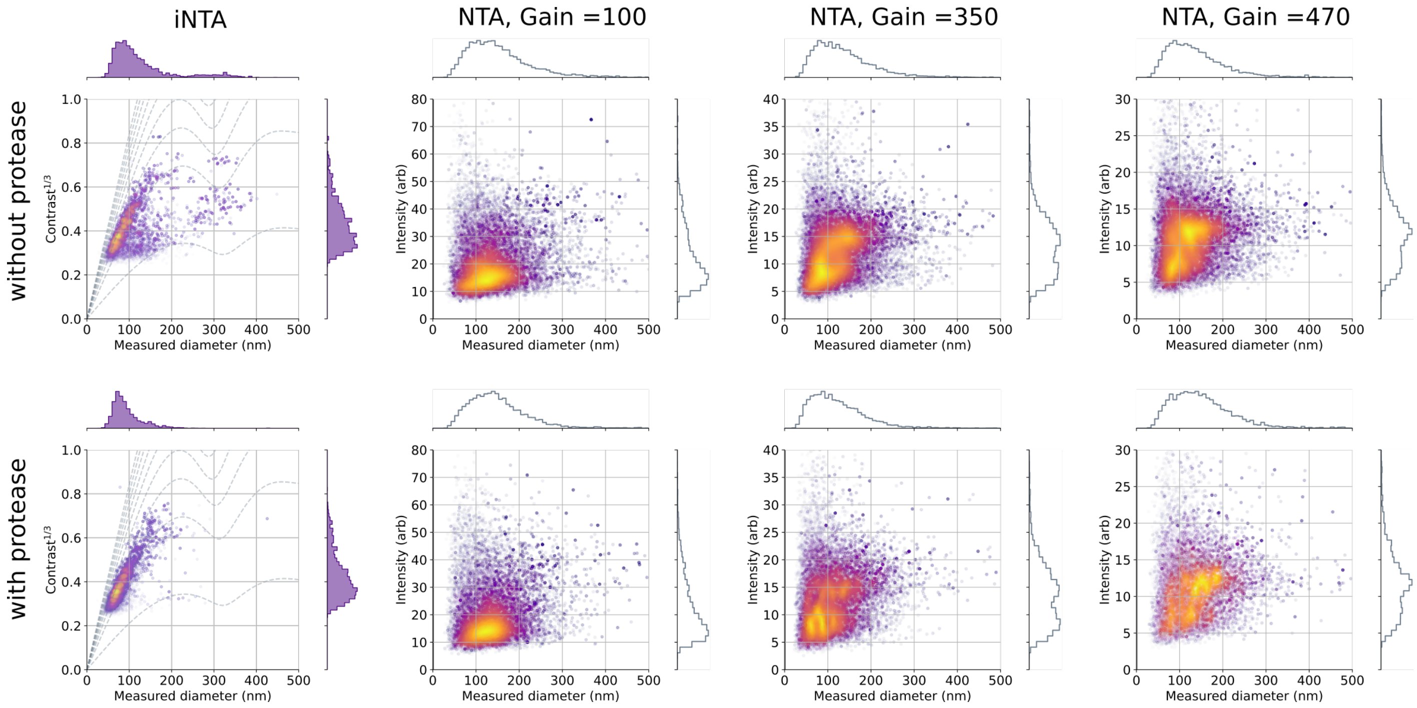

Figure S17: Comparison of urine measurements with iNTA and NTA. (Top row) Urine incubated at 37°C fo 1.5 hours. **(Bottom row)** Urine treated with proteinase K.

References

[1] Silverman, J. M. *et al.* An Exosome-based Secretion Pathway is Responsible for Protein Export from Leishmania and Communication with Macrophages. *J. Cell Sci.* **123**, 842–852 (2010).

[2] Solbach, W., Forberg, K. & Röllinghoff, M. Effect of T-lymphocyte suppression on the parasite burden in Leishmania major-infected, genetically susceptible BALB/c mice. *Infect. Immun.* **54**, 909–912 (1986).

[3] Théry, C. *et al.* Minimal information for studies of extracellular vesicles 2018 (MISEV2018): a position statement of the International Society for Extracellular Vesicles and update of the MISEV2014 guidelines. *Journal of Extracellular Vesicles* **7**, 1535750 (2018).

[4] Bohren, C. & Huffman, D. *Absorption and Scattering of Light by Small Particles* (John Wiley & Sons, Ltd, 1998).

[5] Van Der Pol, E., Coumans, F. A., Sturk, A., Nieuwland, R. & Van Leeuwen, T. G. Refractive index determination of nanoparticles in suspension using nanoparticle tracking analysis. *Nano Lett.* **14**, 6195–6201 (2014).

[6] Khlebtsov, B. N., Khanadeev, V. A. & Khlebtsov, N. G. Determination of the Size, Concentration, and Refractive Index of Silica Nanoparticles from Turbidity Spectra. *Langmuir* **24**, 8964–8970 (2008).

[7] Kashkanova, A. D. *et al.* Precision single-particle localization using radial variance transform. *Opt. Express* **29**, 11070–11083 (2021).

[8] Ashkin, A. Acceleration and Trapping of Particles by Radiation Pressure. *Phys. Rev. Lett.* **24**, 156–159 (1970).

[9] Schätzel, K., Neumann, W.-G., Müller, J. & Materzok, B. Optical tracking of single Brownian particles. *Appl. Optics* **31**, 770 (1992).

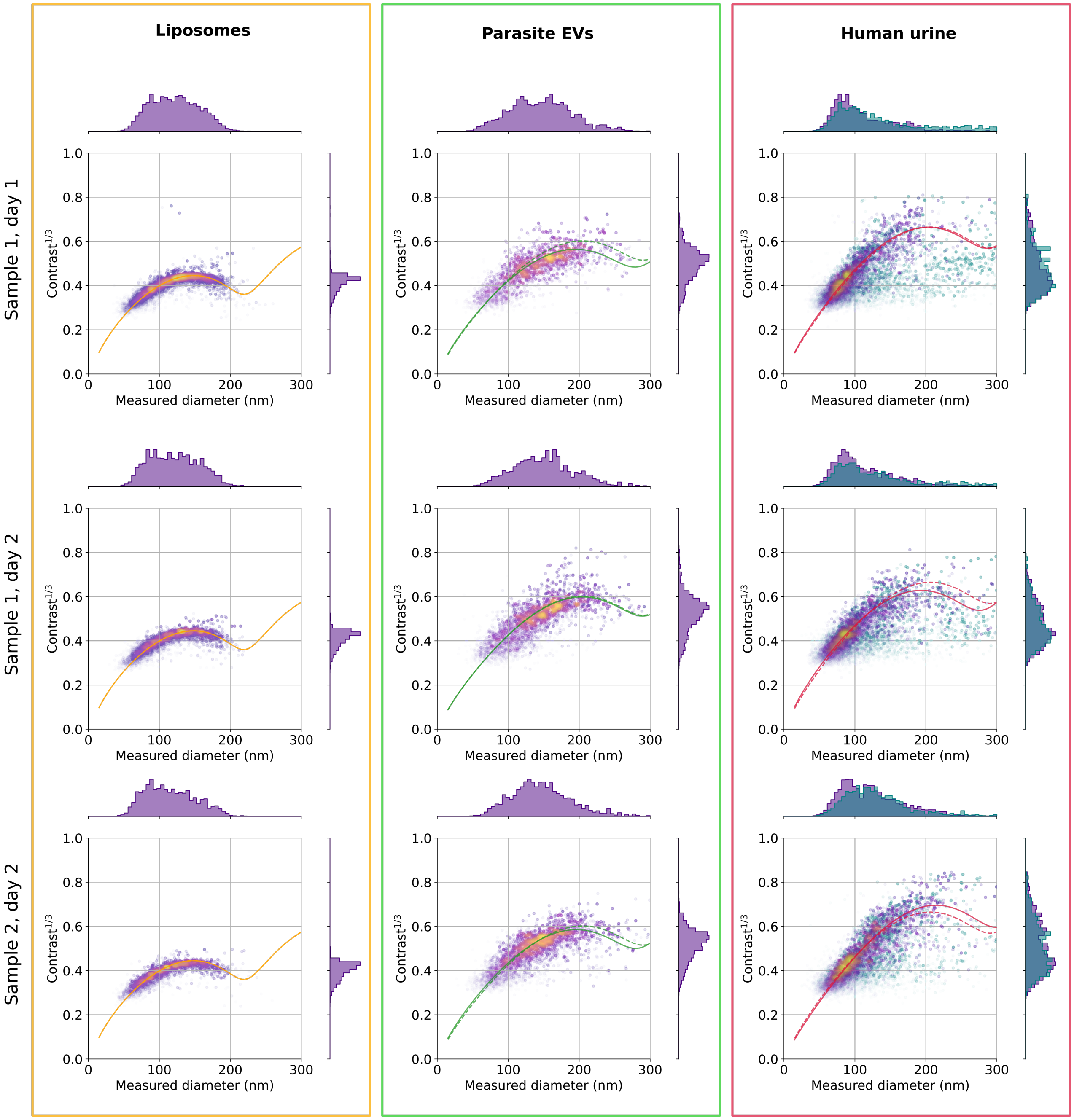

Figure S18: Reproducibility check for biological samples. Each column shows one of the measured sample types. Rows 1 and 2 show measurements of the same sample performed on different days, while rows 2 and 3 show the measurement of different samples prepared on the same day. Solid line indicates the fit to the data, while dashed line indicates the fit to the data shown in the main text.

[10] Allan, D. *et al.* soft-matter/trackpy: Trackpy v0.4.2 (2019).

[11] Michalet, X. Mean square displacement analysis of single-particle trajectories with localization error: Brownian motion in an isotropic medium. *Phys. Rev. E* **82**, 1–13 (2010).

[12] Wagner, T., Lipinski, H.-G. & Wiemann, M. Dark field nanoparticle tracking analysis for size characterization of plasmonic and non-plasmonic particles. *J. Nanopart. Res.* **16** (2014).

[13] Micanovic, R., LaFavers, K., Garimella, P. S., Wu, X.-R. & El-Achkar, T. M. Uromodulin (Tamm–Horsfall protein): guardian of urinary and systemic homeostasis. *Nephrology Dialysis Transplantation* **35**, 33–43 (2019).

[14] Zhu, Y.-Z. *et al.* Significance of palmitoylation of CD81 on its association with tetraspanin-enriched microdomains and mediating hepatitis C virus cell entry. *Virology* **429**, 112–123 (2012).

[15] Jang, D., Kwon, H., Jeong, K., Lee, J. & Pak, Y. Essential role of flotillin-1 palmitoylation in the intracellular localization and signaling function of IGF-1 receptor. *Journal of Cell Science* **128**, 2179–2190 (2015).

[16] Kudo, K. *et al.* Cell surface CD63 increased by up-regulated polylactosamine modification sensitizes human melanoma cells to the BRAF inhibitor PLX4032. *The FASEB Journal* **33**, 3851–3869 (2019).
