# Supplementary material for: Precision size and refractive index analysis of weakly scattering nanoparticles in polydispersions
Source: Nat Methods. 2022 May 9;19(5):586–93. doi: 10.1038/s41592-022-01460-z (PMC9119850; doi:10.1038/s41592-022-01460-z)
Supplement: Supplementary file 2 — Reporting Summary [file 41592_2022_1460_MOESM2_ESM.pdf]

nature portfolio

Corresponding author(s): Vahid Sandoghdar

Last updated by author(s): 15/2/22

Reporting Summary

Nature Portfolio wishes to improve the reproducibility of the work that we publish. This form provides structure for consistency and transparency in reporting. For further information on Nature Portfolio policies, see our Editorial Policies and the Editorial Policy Checklist.

Statistics

For all statistical analyses, confirm that the following items are present in the figure legend, table legend, main text, or Methods section.

n/a	Confirmed	
☐	☒	The exact sample size (*n*) for each experimental group/condition, given as a discrete number and unit of measurement
☐	☒	A statement on whether measurements were taken from distinct samples or whether the same sample was measured repeatedly
☒	☐	The statistical test(s) used AND whether they are one- or two-sided<br>*Only common tests should be described solely by name; describe more complex techniques in the Methods section.*
☒	☐	A description of all covariates tested
☒	☐	A description of any assumptions or corrections, such as tests of normality and adjustment for multiple comparisons
☐	☒	A full description of the statistical parameters including central tendency (e.g. means) or other basic estimates (e.g. regression coefficient) AND variation (e.g. standard deviation) or associated estimates of uncertainty (e.g. confidence intervals)
☒	☐	For null hypothesis testing, the test statistic (e.g. *F*, *t*, *r*) with confidence intervals, effect sizes, degrees of freedom and *P* value noted<br>*Give P values as exact values whenever suitable.*
☒	☐	For Bayesian analysis, information on the choice of priors and Markov chain Monte Carlo settings
☒	☐	For hierarchical and complex designs, identification of the appropriate level for tests and full reporting of outcomes
☒	☐	Estimates of effect sizes (e.g. Cohen's *d*, Pearson's *r*), indicating how they were calculated

Our web collection on statistics for biologists contains articles on many of the points above.

Software and code

Policy information about availability of computer code

Data collection	Video acquisition was performed using a custom pyLabLib Cam-control software developed in our lab and written in Python 3.6 (https://github.com/SandoghdarLab/pyLabLib-cam-control). The software was not developed by the authors of this manuscript, and is not central to the research described here. In principle any video acquisition software can be used, as long as acquisition at high frame rates is supported.
Data analysis	The main data analysis pipeline was written in Python 3.6 and uses freely available standard Python packages as well as imgrvt 1.0.0, pyLabLib 1.2.1, trackpy 0.5.0. It is available in the iNTA repository (https://github.com/SandoghdarLab/iNTA) as version 1.0. It will be incorporated into the PiSCAT package (https://github.com/SandoghdarLab/PiSCAT). In principle any particle tracking software can be used after application of Radial Variance Transform (https://github.com/SandoghdarLab/rvt) to the median background corrected videos. The Mie scattering calculations were performed in Mathematica 10.0, Matlab 2019a and Python 3.6.

For manuscripts utilizing custom algorithms or software that are central to the research but not yet described in published literature, software must be made available to editors and reviewers. We strongly encourage code deposition in a community repository (e.g. GitHub). See the Nature Portfolio guidelines for submitting code & software for further information.

Data

Policy information about availability of data

All manuscripts must include a data availability statement. This statement should provide the following information, where applicable:

- Accession codes, unique identifiers, or web links for publicly available datasets
- A description of any restrictions on data availability
- For clinical datasets or third party data, please ensure that the statement adheres to our policy

An example raw dataset is available in the iNTA repository (https://github.com/SandoghdarLab/iNTA). Due to large size of the individual raw videos, more data are

available from the corresponding author upon request. We have submitted all relevant data of our experiments to the EV-TRACK knowledgebase (EV-TRACK ID: EV220073) (Van Deun J, et al. EV-TRACK: transparent reporting and centralizing knowledge in extracellular vesicle research. Nature methods. 2017;14(3):228-32).

Field-specific reporting

Please select the one below that is the best fit for your research. If you are not sure, read the appropriate sections before making your selection.

☒ Life sciences ☐ Behavioural & social sciences ☐ Ecological, evolutionary & environmental sciences

For a reference copy of the document with all sections, see nature.com/documents/nr-reporting-summary-flat.pdf

Life sciences study design

All studies must disclose on these points even when the disclosure is negative.

Sample size	We measure nanoparticles diffusing in solutions and each presented histogram contains on the order of 1000 trajectories and 1e6 localization with details for each individual measurement given in the Methods section. This number of trajectories and localizations was a result of keeping measurement time reasonable (on the order of several minutes) for dilute samples.
Data exclusions	Only trajectories with more than 25 localizations were kept to decrease the error in diffusion constant determination.
Replication	The measurements are very reproducible. We verified that the measured hydrodynamic size does not depend on the incident laser power, camera chip illumination, and the focal plane position. However as indicated in the inset of figure 2b, temperature and viscosity have an effect on the extracted diffusion constant and therefore need to be controlled. Additionally the buffer properties (pH, salt concentration) could have an effect on the hydration layer and therefore measured particle size. As is now shown in the SI selected measurements were repeated 3-20 times to ensure repeatability.
Randomization	Not applicable. The measurement was by default performed on a random sample of particles, which diffuse into the illumination volume. The necessary amount of solution was also selected randomly with a pipette.
Blinding	Not required, since all the samples of the same kind (e.g. all biological samples) were analyzed with the same exact code without modifications. In that sense the analysis is "blind".

Reporting for specific materials, systems and methods

We require information from authors about some types of materials, experimental systems and methods used in many studies. Here, indicate whether each material, system or method listed is relevant to your study. If you are not sure if a list item applies to your research, read the appropriate section before selecting a response.

Materials & experimental systems

n/a	Involved in the study	
☐	☒	Antibodies
☒	☐	Eukaryotic cell lines
☒	☐	Palaeontology and archaeology
☐	☒	Animals and other organisms
☐	☒	Human research participants
☒	☐	Clinical data
☒	☐	Dual use research of concern

Methods

n/a	Involved in the study	
☒	☐	ChIP-seq
☒	☐	Flow cytometry
☒	☐	MRI-based neuroimaging

Antibodies

Antibodies used

Primary antibodies

anti-TSG101: Proteintech [Manchester, UK]; 28283-1-AP; NaN; 00080154
anti-Flotilin-1: Proteintech [Manchester, UK]; 15571-1-AP; NaN; 00053543
anti-Uromodulin: Proteintech [Manchester, UK]; 11911-1-AP; NaN; 00056125
anti-CD63: Proteintech [Manchester, UK]; 67605-1-Ig; 3D4D1; 10015952
anti-CD81: Proteintech [Manchester, UK]; 66866-1-Ig; 1G2C6; 10017531 & 10011817
anti-Alix: Proteintech [Manchester, UK]; 67715-1-Ig; 1H9D9; 10017670

Secondary antibodies

Goat Anti-Mouse IgG (H+L)-HRP Conjugate; Bio-Rad Laboratories [Feldkirchen, GER]; #170-6516; 64322731
Goat Rabbit-Mouse IgG (H+L)-HRP Conjugate; Bio-Rad Laboratories [Feldkirchen, GER]; #170-6515; 64332296

Validation	See the attached Antibody_validation.pdf

Animals and other organisms

Policy information about studies involving animals; ARRIVE guidelines recommended for reporting animal research

Laboratory animals	The study did not involve laboratory animals
Wild animals	The study did not involve wild animals
Field-collected samples	Leishmania exosomes were purified from axenic culture of Leishmania major promastigotes (strain MHOM/IL/81/ FEBNI)
Ethics oversight	No ethical approval or guidance was required, since Leishmania promastigotes are not live vertebrates.

Note that full information on the approval of the study protocol must also be provided in the manuscript.

Human research participants

Policy information about studies involving human research participants

Population characteristics	One white male, 30 years old
Recruitment	Only one volunteer was recruited. This prevents us from making general statements about human urine, beyond this particular sample. However, a single sample was sufficient to demonstrate the advantages of iNTA.
Ethics oversight	The Ethics Committee of the Friedrich Alexander University Erlangen waived the need for ethics approval. The donor provided written informed consent prior to enrolment in the study.

Note that full information on the approval of the study protocol must also be provided in the manuscript.
